# Male-Killing *Spiroplasma* Induces Sex-Specific Cell Death via Host Apoptotic Pathway

**DOI:** 10.1371/journal.ppat.1003956

**Published:** 2014-02-13

**Authors:** Toshiyuki Harumoto, Hisashi Anbutsu, Takema Fukatsu

**Affiliations:** Bioproduction Research Institute, National Institute of Advanced Industrial Science and Technology (AIST), Tsukuba, Japan; Stanford University, United States of America

## Abstract

Some symbiotic bacteria cause remarkable reproductive phenotypes like cytoplasmic incompatibility and male-killing in their host insects. Molecular and cellular mechanisms underlying these symbiont-induced reproductive pathologies are of great interest but poorly understood. In this study, *Drosophila melanogaster* and its native *Spiroplasma* symbiont strain MSRO were investigated as to how the host's molecular, cellular and morphogenetic pathways are involved in the symbiont-induced male-killing during embryogenesis. TUNEL (terminal deoxynucleotidyl transferase dUTP nick end labeling) staining, anti-cleaved-Caspase-3 antibody staining, and apoptosis-deficient mutant analysis unequivocally demonstrated that the host's apoptotic pathway is involved in *Spiroplasma*-induced male-specific embryonic cell death. Double-staining with TUNEL and an antibody recognizing epidermal marker showed that embryonic epithelium is the main target of *Spiroplasma*-induced male-specific apoptosis. Immunostaining with antibodies against markers of differentiated and precursor neural cells visualized severe neural defects specifically in *Spiroplasma*-infected male embryos as reported in previous studies. However, few TUNEL signals were detected in the degenerate nervous tissues of male embryos, and the *Spiroplasma*-induced neural defects in male embryos were not suppressed in an apoptosis-deficient host mutant. These results suggest the possibility that the apoptosis-dependent epidermal cell death and the apoptosis-independent neural malformation may represent different mechanisms underlying the *Spiroplasma*-induced male-killing. Despite the male-specific progressive embryonic abnormality, *Spiroplasma* titers remained almost constant throughout the observed stages of embryonic development and across male and female embryos. Strikingly, a few *Spiroplasma*-infected embryos exhibited gynandromorphism, wherein apoptotic cell death was restricted to male cells. These observations suggest that neither quantity nor proliferation of *Spiroplasma* cells but some *Spiroplasma*-derived factor(s) may be responsible for the expression of the male-killing phenotype.

## Author Summary

Symbiotic bacteria are ubiquitously associated with diverse insects, and affect their host biology in a variety of ways. In *Drosophila* fruit flies, infection with *Spiroplasma* symbionts often causes male-specific embryonic mortality, resulting in the production of all-female offspring. This striking phenotype is called “male-killing”, whose underlying mechanisms are of great interest. Here we investigated *Drosophila melanogaster* and its native *Spiroplasma* symbiont strain to understand how the host's molecular, cellular and morphogenetic pathways are involved in the symbiont-induced male-killing. Specifically in *Spiroplasma*-infected male embryos, pathogenic phenotypes including massive cell death throughout the body and neural malformation were observed. We unequivocally identified that the male-specific cell death preferentially occurs in the embryonic epithelium via the host's apoptotic pathway. Meanwhile, we found that, unexpectedly, the male-specific neural defects occur independently of host's apoptosis, suggesting that at least two different mechanisms may be involved in the *Spiroplasma*-induced male-killing. Also unexpected was the finding that *Spiroplasma* titers are almost constant throughout embryogenesis irrespective of sex despite the male-specific severe apoptosis. We serendipitously found *Spiroplasma*-infected sexual mosaic embryos, wherein apoptosis was associated with male cells, which suggests that some *Spiroplasma*-derived factor(s) may selectively act on male cells and cause male-killing.

## Introduction

Symbiotic microorganisms are ubiquitously associated with diverse insects, and affect their host biology in a variety of ways [1], [2]. Some symbionts play important biological roles such as provisioning of essential nutrients to their hosts [3], helping food digestion for their hosts [4], or improving the fitness of their hosts under specific ecological conditions [5]. Other symbionts like *Wolbachia*, *Cardinium* and *Spiroplasma* are generally parasitic rather than beneficial to their hosts, often causing negative fitness effects and also inducing reproductive phenotypes like cytoplasmic incompatibility, male-killing, parthenogenesis or feminization, by which these symbionts are able to spread their own infections into the host populations in a selfish manner [6]–[8].

Members of the genus *Spiroplasma*, belonging to the class *Mollicutes*, are wall-less bacteria associated with diverse arthropods and plants [9]. Some *Spiroplasma* species and strains are known to cause male-killing phenotypes in fruit flies, ladybird beetles and butterflies, wherein infected females produce all-female or female-biased offspring due to male-specific mortality during embryogenesis and/or larval development [10], [11]. Male-killing symbiotic bacteria belonging to *Spiroplasma poulsonii* [12] have been identified from fruit flies of the genus *Drosophila*, which are represented by the strains WSRO from *D. willistoni*, NSRO from *D. nebulosa*, MSRO from *D. melanogaster* and others [13], [14].

While the *Drosophila*-*Wolbachia* symbiosis represents one of the best-studied model symbiotic systems [8], [15], the *Drosophila*-*Spiroplasma* symbiosis has also been well-studied as another model system of infection dynamics [16]–[18], immune regulation [19]–[21], vertical transmission [22], [23] and male-killing expression [24]–[27]. However, molecular and cellular mechanisms underlying the *Spiroplasma*-induced male-specific embryonic pathology are still not well understood. Histological observations, mosaic analysis and *in vitro* culturing have suggested that nervous system is among the major target sites of *Spiroplasma*-induced male-killing in *Drosophila* embryos [28]–[32]. In *D. melanogaster*, *Spiroplasma*-infected mutants deficient in dosage compensation complex genes fail to show male-killing phenotype, indicating that a functional dosage compensation complex is required for expression of the *Spiroplasma*-induced make-killing [24]. In *D. nebulosa* infected with its native *Spiroplasma* strain NSRO, dying male embryos exhibit widespread TUNEL (terminal deoxynucleotidyl transferase dUTP nick end labeling) signals, suggesting possible involvement of host's pathway of programmed cell death or apoptosis [25].

In this study, we performed detailed investigation of the male-killing process during embryogenesis of *D. melanogaster* infected with its native *Spiroplasma* strain MSRO. In particular, we focused on host's molecular, cellular and morphogenetic pathways that may potentially be involved in the male-killing phenotype by utilizing the wealth of genetic resources available in *D. melanogaster*. Our observations unveiled several previously unknown aspects of *Spiroplasma*-induced male-killing, which include unequivocal demonstration of male-specific up-regulation of apoptotic pathway, identification of embryonic epithelium as the main target of male-specific apoptosis, male-specific malformation of embryonic nervous system independent of apoptosis, and specific killing of male cells in gynandromorphic embryos.

## Results and Discussion

### TUNEL-based detection of ectopic programmed cell death in *Spiroplasma*-infected male embryos

**Figure 1:**
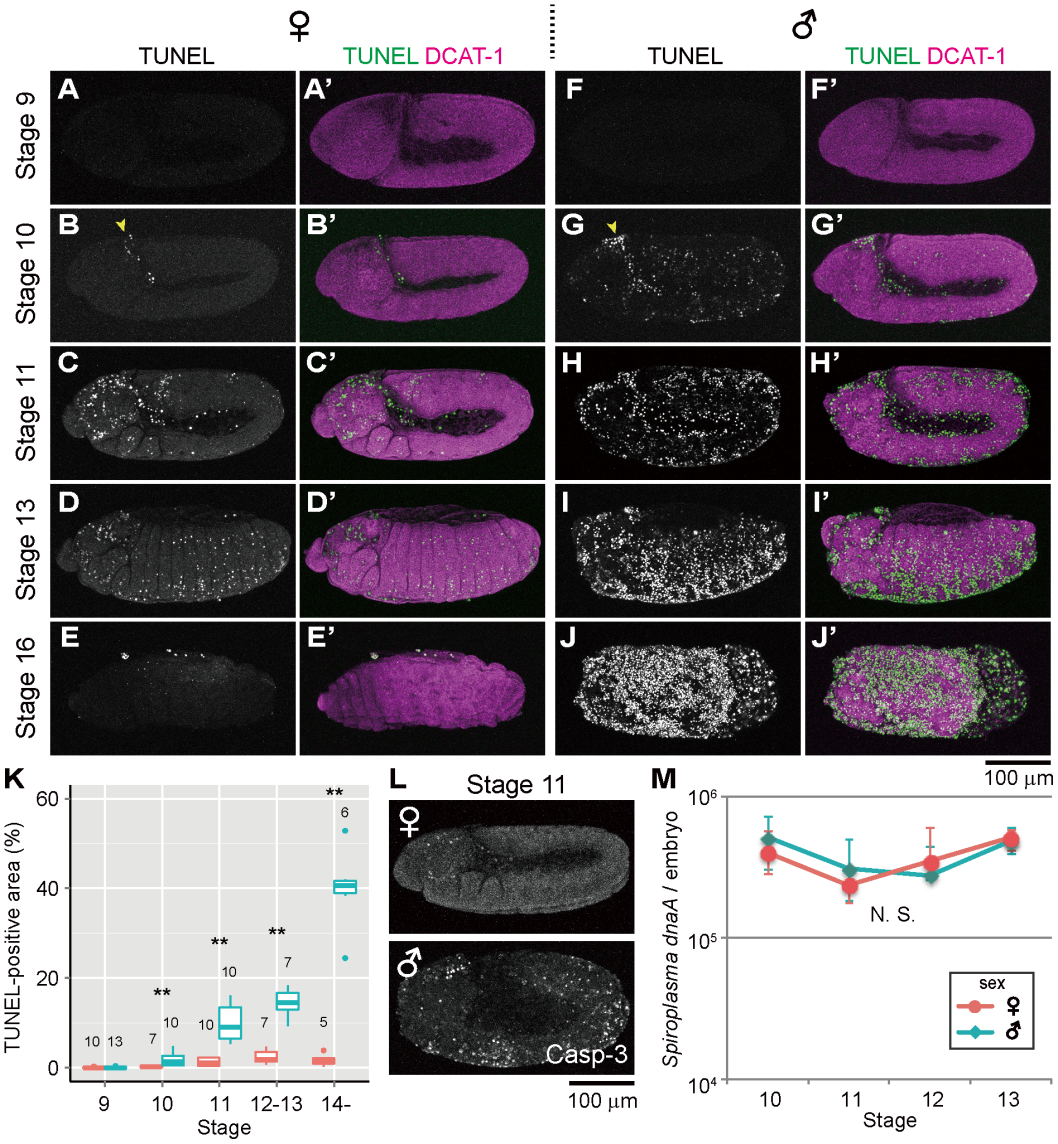
Ectopic cell death in *Spiroplasma*-infected male embryos of *D. melanogaster*. (**A**–**E**) *Spiroplasma*-infected female embryos and (**F**–**J**) *Spiroplasma*-infected male embryos stained with TUNEL and an antibody against *Drosophila* α-Catenin (DCAT-1) for visualization of embryonic morphology. Developmental stages are indicated on the left side. Yellow arrowheads indicate the cephalic region at stage 10 where TUNEL positive cells appear first. (**K**) Quantification of TUNEL-positive area in *Spiroplasma*-infected female embryos (red) and male embryos (blue). Medians and interquartile ranges are shown with sample sizes (Wilcoxon rank sum test; **, *P*<0.01). (**L**) Anti-cleaved Caspase-3 antibody (Casp-3) staining of *Spiroplasma*-infected female and male embryos at stage 11. (**M**) Infection dynamics of the male-killing *Spiroplasma* in developing female embryos (red) and male embryos (blue) from stage 10 to stage 13. Infection densities are indicated in terms of symbiont *dnaA* gene copies per embryo. Medians and interquartile ranges of 12 measurements are shown. No significant difference was observed between sexes or stages (Kruskal-Wallis test followed by Scheffe test; *P*>0.05).

In *Spiroplasma*-infected female embryos (sexed according to *Sex-lethal* [*Sxl*] expression, see Materials and Methods and Fig. S1A and B), TUNEL-positive cells were scarcely found by stage 9 (Fig. 1A), and first appeared at stage 10 in the cephalic region (Fig. 1B, arrowhead). Subsequently, TUNEL-labeled cells spread to the other regions (Fig. 1C and D) and reached a peak level at stages 12–13 (Fig. 1K). These patterns are typical of normal programmed cell death in *Drosophila* development [33]. Actually, the *Spiroplasma*-infected female embryos exhibited no substantial differences in the spatiotemporal appearance of TUNEL-positive cells in comparison with uninfected male and female embryos (Fig. 1C; Fig. S1C–E). The *Spiroplasma*-infected female embryos developed normally (Fig. 1E), and finally emerged as first instar larvae. By contrast, in *Spiroplasma*-infected male embryos, ectopic TUNEL signals were observed at stage 10: in addition to the dense signals at the cephalic region (Fig. 1G, arrowhead), TUNEL-positive cells were detected throughout the embryonic body (Fig. 1G). Subsequently, the excessive TUNEL signals became more prominent and progressively increased during germ band retraction (Fig. 1H, I and K). From stage 13 and on, the *Spiroplasma*-infected male embryos started to disintegrate with massive cell death, wherein segmentation and other morphological traits of the embryos became difficult to recognize (Fig. 1J), and finally died. These results indicate that *Spiroplasma*-infected *Drosophila* males exhibit ectopic programmed cell death from the early stage of embryonic development. A previous study reported that, in *D. nebulosa* and its natural *Spiroplasma* strain NSRO, *Spiroplasma*-infected male embryos exhibit developmental arrest between stages 12 and 13 with segmentation failure, disintegrated embryonic morphology, and widespread apoptosis as testified by TUNEL staining [25]. Our observations with *D. melanogaster* and its natural *Spiroplasma* strain MSRO are highly concordant with the previous observations, suggesting that the same molecular and cellular processes are operating under the symbiont-induced male-specific cell death in the different host species.

### TUNEL-independent detection of ectopic programmed cell death in *Spiroplasma*-infected male embryos

Previous studies have demonstrated that normal programmed cell death in *Drosophila* development requires the activity of Caspase-9-like initiator caspase Dronc (*Drosophila* Nedd2-like caspase) [34]–[38], and an antibody against cleaved-Caspase-3 can detect the Dronc activity [39]. When probed with the anti-cleaved-Caspase-3 antibody, *Spiroplasma*-infected male embryos exhibited more immunopositive signals than *Spiroplasma*-infected female embryos as well as uninfected male and female embryos, and the spatiotemporal patterns of the signals looked similar to those of the TUNEL signals (Fig. 1L). These results suggest that *Spiroplasma*-infected *Drosophila* males exhibit ectopic programmed cell death during embryonic development, at least in part by activating the caspase-dependent apoptotic pathway.

### Population dynamics of *Spiroplasma* in *Drosophila* embryos

*Spiroplasma*-infected male embryos and female embryos were individually subjected to quantitative PCR targeting *Spiroplasma dnaA* gene copies. Throughout the embryonic stages examined (from 10 to 13), *Spiroplasma* titers per embryo remained almost constant, exhibiting no significant differences between male embryos and female embryos (Fig. 1M). By contrast, *Spiroplasma* titers per host *elongation factor 1α 100E* (*EF1a*) gene copy exhibited higher values in male embryos than in female embryos (Fig. S1F), which was attributable to lower *EF1α* gene titers in male embryos presumably because of male-killing phenotype (Fig. S1G). These results strongly suggest that the ectopic programmed cell death specific to male embryos entails no *Spiroplasma* proliferation during embryogenesis. Meanwhile, it should be noted that, since quantitative PCR detects not live bacterial cells but DNA molecules, the possibility cannot be ruled out that titers of live *Spiroplasma* cells may change during embryogenesis and/or between male embryos and female embryos.

### Host's apoptotic pathway is involved in ectopic cell death in *Spiroplasma*-infected male embryos

**Figure 2:**
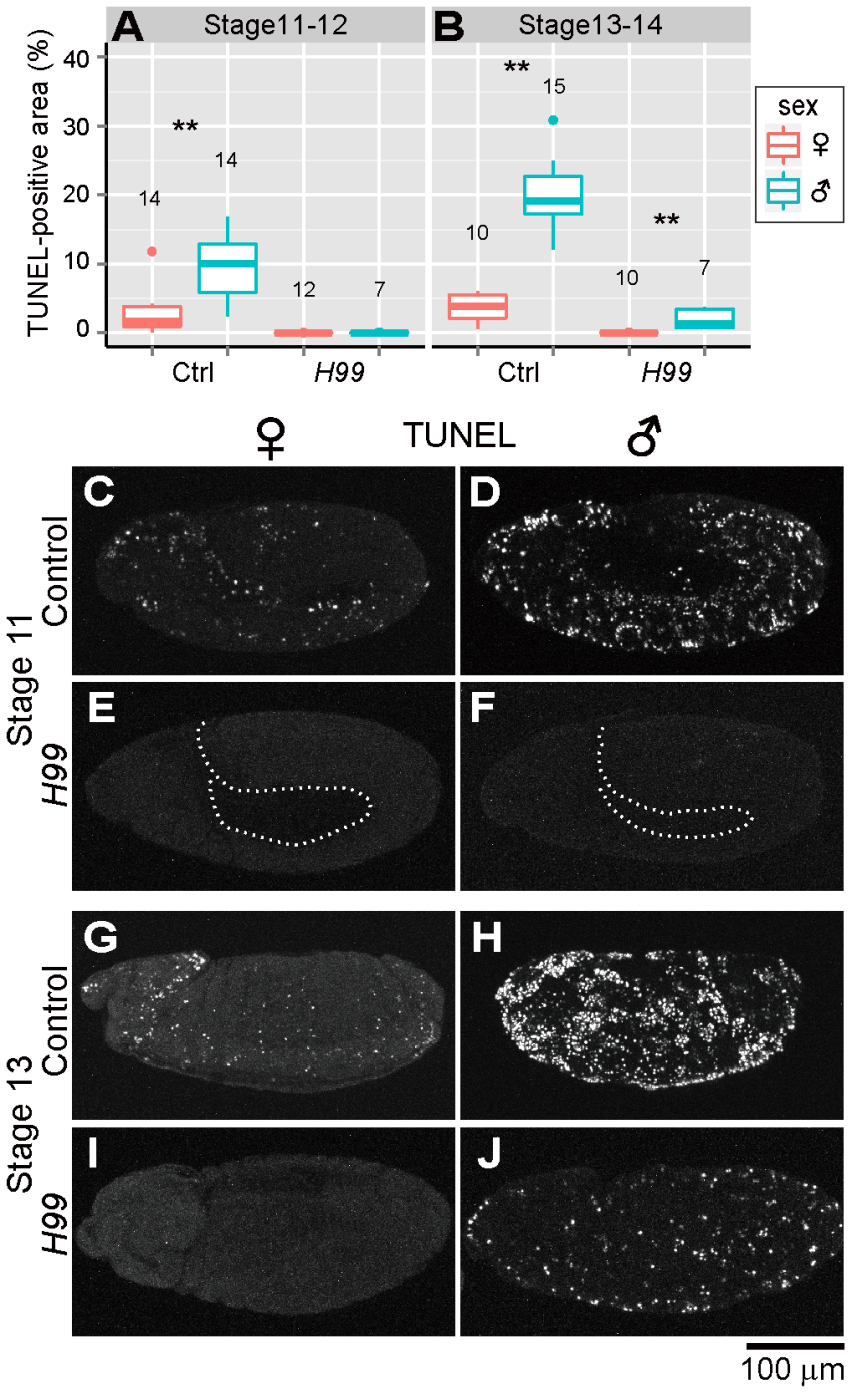
Involvement of host's apoptotic pathway in ectopic cell death in *Spiroplasma*-infected male embryos of *D. melanogaster*. (**A** and **B**) Quantification of TUNEL-positive area per embryo in control embryos (Ctrl: *H99/+* genotype) and apoptosis-deficient embryos (*H99*: *H99/H99* genotype) at stage 11–12 (**A**) and stage 13–14 (**B**) (Wilcoxon rank sum test; **, *P*<0.01). (**C**–**J**) TUNEL signals in the *Spiroplasma*-infected control embryos and apoptosis-deficient embryos at stage 11 (**C**–**F**) and stage 13 (**G**–**J**). The internal edge of the epidermis is highlighted by a dashed line in (**E**) and (**F**).

Previous studies have demonstrated that programmed cell death in normal *Drosophila* development requires proapoptotic genes *reaper* (*rpr*), *head involution defective* (*hid*) and *grim*, which are collectively termed RHG genes [40]–[42]. In *Drosophila* mutant *H99* deficient in all these genes, apoptotic cell death is almost completely blocked during embryogenesis [40]. RHG proteins bind to *Drosophila* inhibitor of apoptosis protein 1 (DIAP1) and disrupt its ability to inhibit caspase activity, by which apoptosis is triggered [43]–[46]. When *Spiroplasma*-infected *H99* mutant embryos were subjected to the TUNEL assay, TUNEL-positive cells were observed neither in female embryos nor in male embryos at stages 11 and 12 (Fig. 2A and C–F). These results strongly suggest that the majority of the ectopic programmed cell death observed in *Spiroplasma*-infected male embryos is induced via host's apoptotic pathway.

### Residual ectopic cell death in *Spiroplasma*-infected and apoptosis-suppressed male embryos

In later stages of embryogenesis (stages 13 and 14), some TUNEL-positive cells were detected specifically in *Spiroplasma*-infected *H99* male embryos, although the level of the signals was significantly lower than that in *Spiroplasma*-infected control male embryos (Fig. 2B and G–J). The residual TUNEL-positive cells may implicate the presence of a minor pathway of *Spiroplasma*-induced male-specific cell death independent of RHG proteins. Alternatively, the residual TUNEL-positive cells may be due to attenuated clearance of dead cells in the infected embryos.

### Host's Hox genes and segment polarity genes are not involved in ectopic cell death in *Spiroplasma*-infected male embryos

One of the major mechanisms of apoptosis regulation in *Drosophila* development is the expression control of RHG genes [44], [47]. Notably, it was reported that several Hox proteins, such as Deformed (Dfd) and Abdominal B (Abd-B), may directly activate the expression of *rpr* by binding to its enhancer elements [48]. It was also reported that some segment polarity genes may regulate cell survival and death to establish morphological patterns during embryogenesis [49]. However, when we performed immunohistochemical visualization of Hox proteins Antennapedia (Antp), Ultrabithorax (Ubx) and Abd-B, and several segment polarity proteins Wingless (Wg) and Engrailed (En) in *Spiroplasma*-infected male embryos and female embryos, no sex-related differences were observed in their localization patterns (Fig. S2). These results refute the possibility that *Spiroplasma* infection may induce ectopic cell death by affecting such developmental signals as Hox genes and segment polarity genes in male embryos.

### Ectopic cell death occurs in epithelial cells of *Spiroplasma*-infected male embryos

**Figure 3:**
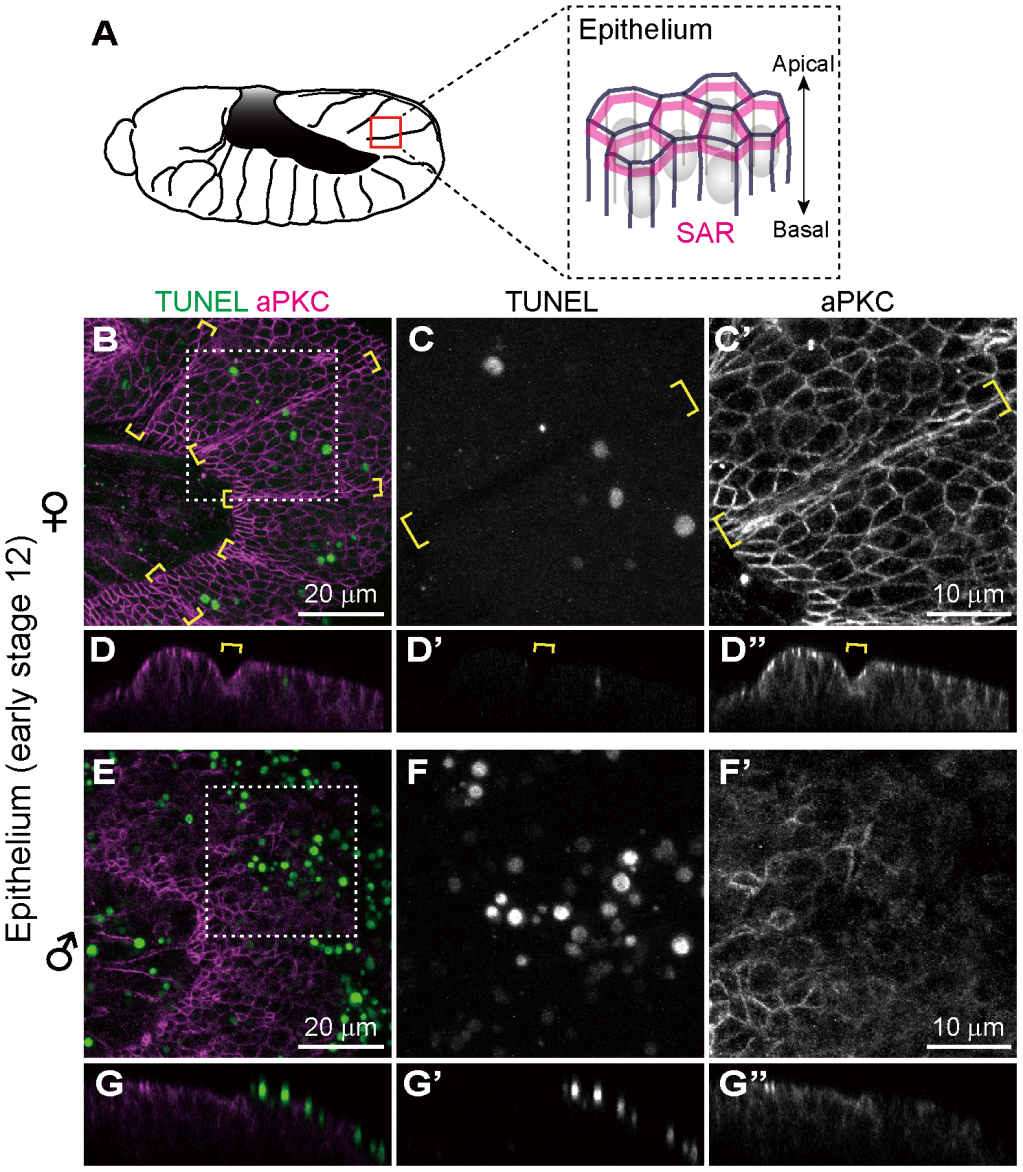
Ectopic cell death in epithelial cells of *Spiroplasma*-infected male embryos of *D. melanogaster*. (**A**) Schematic diagram of epithelial constitution at early stage 12 when segmentation of embryonic body becomes evident. Each epithelial cell develops a distinct apical-basal polarity. A portion of the apical membrane constitutes the subapical region (SAR; magenta), which shares molecular similarities with the vertebrate tight junction [50]. (**B**–**G**) *Spiroplasma*-infected female embryos (**B**–**D**) and male embryos (**E**–**G**) at early stage 12 stained with TUNEL and anti-aPKC antibody that visualizes the SAR of epithelial cells. The boxed regions in (**B**) and (**E**) are magnified in (**C**) and (**F**), respectively. Z-sections of (**B**) and (**E**) are shown in (**D**) and (**G**), respectively. Intersegmental furrows are indicated by yellow brackets.

In *Drosophila*, atypical protein kinase C (aPKC) localizes to the subapical region (SAR) in the epithelial junctions, thereby establishing apical-basal cell polarity (Fig. 3A) [50], [51]. We visualized embryonic epithelial cells by immunostaining with anti-aPKC antibody. In *Spiroplasma*-infected female embryos, aPKC showed normal junctional localization and subapical localization in epithelial cells (Fig. 3B–D). In *Spiroplasma*-infected male embryos, by contrast, junctional localization of aPKC was significantly impaired (Fig. 3E and F), and concentrated TUNEL signals were observed at the level of the subapical region of epithelial cells (Fig. 3G). Notably, intersegmental furrows, which were normally formed in *Spiroplasma*-infected female embryos (Fig. 3B–D, brackets), became obscure in *Spiroplasma*-infected male embryos (Fig. 3E–G). These results indicate that embryonic epithelium is among the target tissues wherein *Spiroplasma*-associated male-specific programmed cell death is induced. *Spiroplasma*-infected male embryos show ambiguous segments after germ band retraction and die (Fig. 1I) [25]. The loss of segmentation may be relevant to the epithelial damage due to the male-specific apoptosis.

### Ectopic cell death does not occur in differentiated neural cells of *Spiroplasma*-infected male embryos

**Figure 4:**
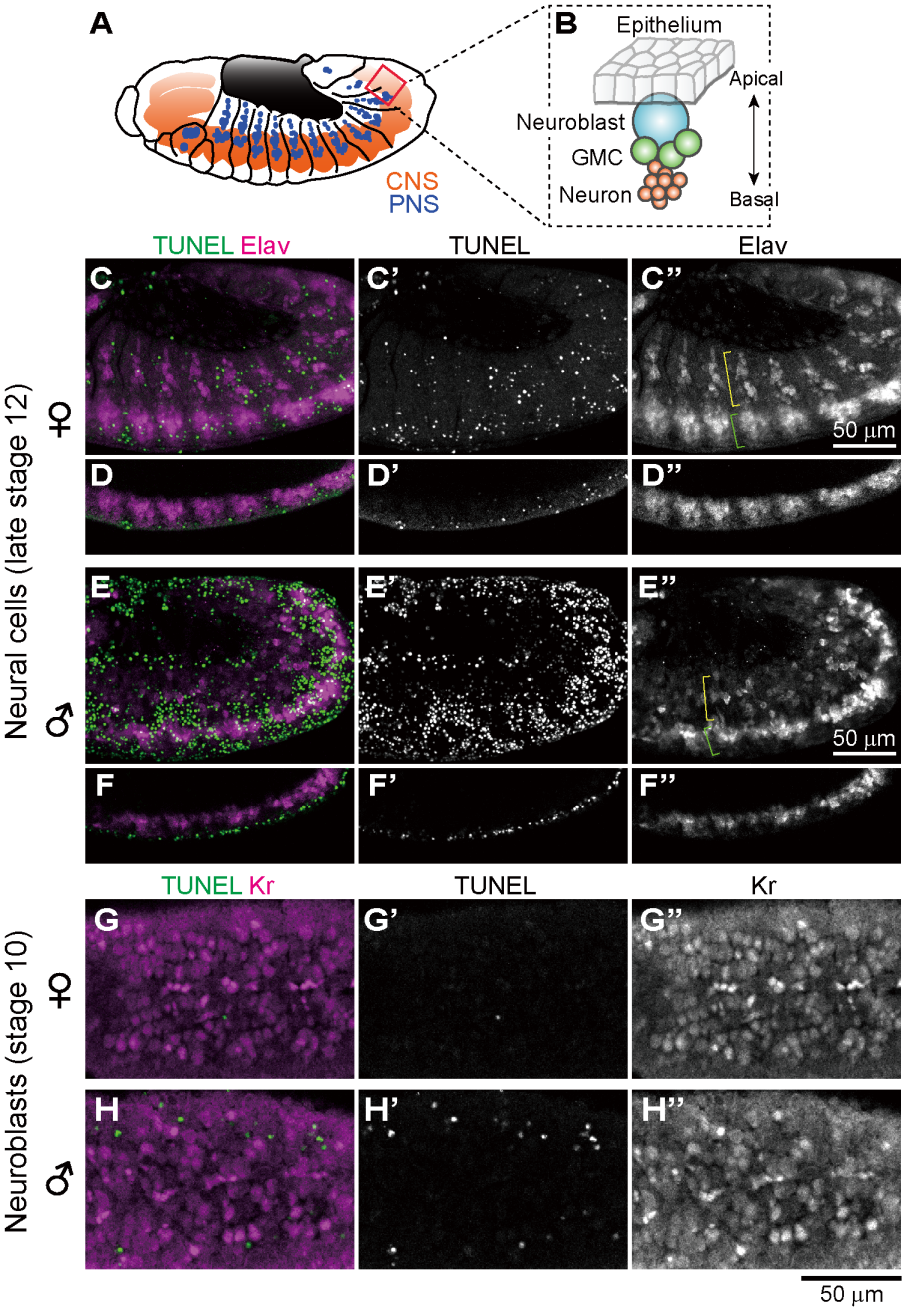
Ectopic cell death and neural defects in *Spiroplasma*-infected male embryos of *D. melanogaster*. (**A**) Schematic diagram of the embryonic nervous system at late stage 12, wherein the central nervous system (CNS) and the peripheral nervous system (PNS) are depicted in red and blue, respectively. (**B**) Schematic diagram of neurogenesis at the cellular level: blue, neuroblast (or precursor neural cell); green, ganglion mother cells (or GMC); and red, neurons (or differentiated neural cells). (**C**–**F**) Lateral views of *Spiroplasma*-infected female embryos (**C**–**D**) and male embryos (**E**–**F**) at late stage 12 stained with TUNEL and anti-Elav antibody that visualizes differentiated neural cells. Single optical-sections through CNS of (**C**) and (**E**) are shown in (**D**) and (**F**), respectively. Green brackets and yellow brackets indicate CNS and PNS, respectively. (**G**–**H**) Dorsal views of *Spiroplasma*-infected female embryos (**G**) and male embryos (**H**) at stage 10 stained with TUNEL and anti-Krüppel (Kr) antibody that visualizes precursor neural cells or neuroblasts. Single optical sections through the neuroblast layer are selected.

Previous histological observations, mosaic analysis and *in vitro* culturing have suggested that nervous system is among the major target sites of *Spiroplasma*-induced male-killing in *Drosophila* embryos [28]–[32]. In *Drosophila* embryos, Elav (embryonic lethal abnormal vision) protein is specifically expressed in differentiated neural cells [52], [53]. We performed immunostaining of developing embryos with anti-Elav antibody, which clearly visualized neurons in central nervous system (CNS) and also neurons in peripheral nervous system (PNS) (Fig. 4A). In *Spiroplasma*-infected male embryos, both CNS and PNS were disorganized (Fig. 4E and F), whereas these neural structures were intact in *Spiroplasma*-infected female embryos (Fig. 4C and D). It is noteworthy that, despite the remarkable structural disorder, the regions of concentrated TUNEL-positive cells in *Spiroplasma*-infected male embryos did not agree with the locations of CNS and PNS (Fig. 4E and F). These results suggest that *Spiroplasma* infection certainly disrupts the formation of normal nervous system specifically in male embryos, but the neural defects are not due to apoptosis of already differentiated neural cells.

### Ectopic cell death does not occur in precursor neural cells of *Spiroplasma*-infected male embryos

In *Drosophila* embryos, precursor neural cells, or neuroblasts, delaminate basally from neuroectoderm and undergo asymmetric cell division to generate neuroblast itself and ganglion mother cells (GMC). GMCs divide once to give rise to two neurons (Fig. 4B). Neuroblasts express several transcription factors sequentially to generate diverse populations of neurons, one of which is the transcription factor Krüppel (Kr) [54]. *Spiroplasma*-infected embryos at stage 10 were immunostained with anti-Kr antibody to observe neuroblasts at the beginning of male-specific ectopic cell death. Numerous Kr signals were identified in the neuroblast layer of both *Spiroplasma*-infected female embryos and male embryos, but they did not co-localize with the TUNEL signals (Fig. 4G and H). These results suggest that *Spiroplasma* infection disrupts the formation of normal nervous system specifically in male embryos, but the neural defects are unlikely due to direct killing of precursor neural cells via apoptosis.

### *Spiroplasma*-infected male embryos exhibit neural malformation even when apoptosis is suppressed

**Figure 5:**
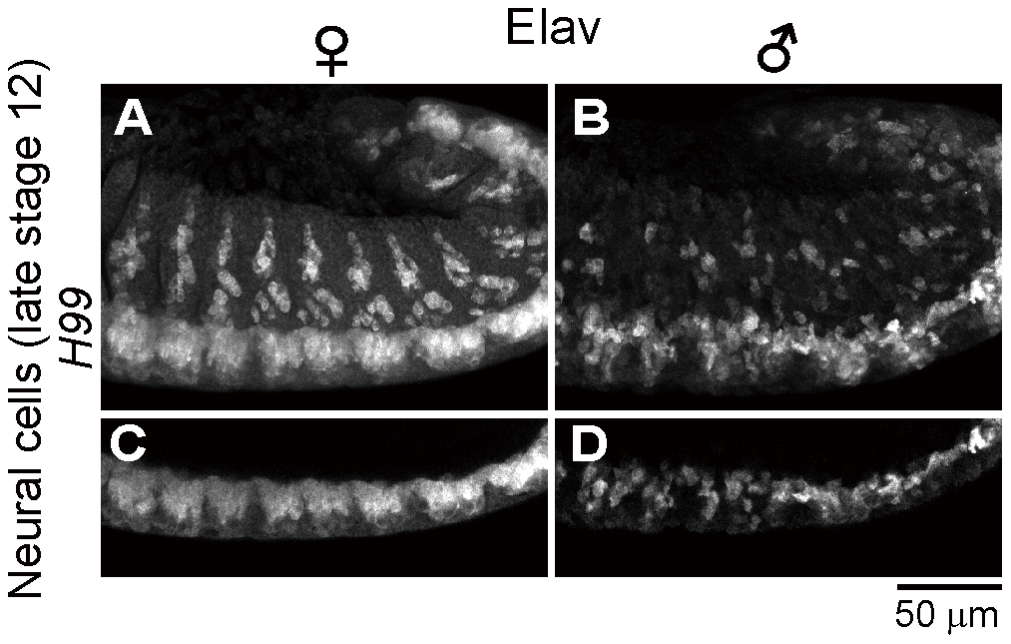
Neural defects in *Spiroplasma*-infected male embryos of apoptosis-deficient mutant of *D. melanogaster*. *Spiroplasma*-infected female embryos (**A** and **C**) and male embryos (**B** and **D**) of the apoptosis-deficient *H99/H99* genotype at late stage 12 stained with anti-Elav antibody to visualize CNS and PNS. Single optical-sections through CNS of (**A**) and (**B**) are shown in (**C**) and (**D**), respectively.

In the light of these results, it is of focal interest how the epithelial apoptosis and the neural malformation in the *Spiroplasma*-infected male embryos are interconnected to each other. Are there any causal relationships between them, or do they represent independent processes leading to the embryonic male lethality? To address this question, we observed the development of nervous system in *Spiroplasma*-infected *H99* mutant embryos, in which apoptotic cell death is almost completely blocked [40], by immunostaining with anti-Elav antibody. In *Spiroplasma*-infected *H99* female embryos, CNS and PNS developed normally (Fig. 5A and C), while in *Spiroplasma*-infected *H99* male embryos, remarkable neural malformation was observed (Fig. 5B and D). These results strongly suggest that host's apoptotic pathway is not required for expression of the neural malformation in *Spiroplasma*-infected male embryos. Plausibly, the male-specific neural malformation may occur independently of the male-specific epithelial apoptosis in the *Spiroplasma*-infected embryos. If so, the *Spiroplasma*-induced male-killing entails at least two independent mechanisms: one targets male epithelial cells via host's apoptotic pathway and another targets male nervous system via unknown pathway(s). Alternatively, the defects in neural tissues may somehow influence the organization of adjacent epithelial cells, thereby causing the male-specific epithelial apoptosis secondarily, or *vise versa*.

### Identification of gynandromorphic *Spiroplasma*-infected embryos, and male-specific cell death within the embryos

**Figure 6:**
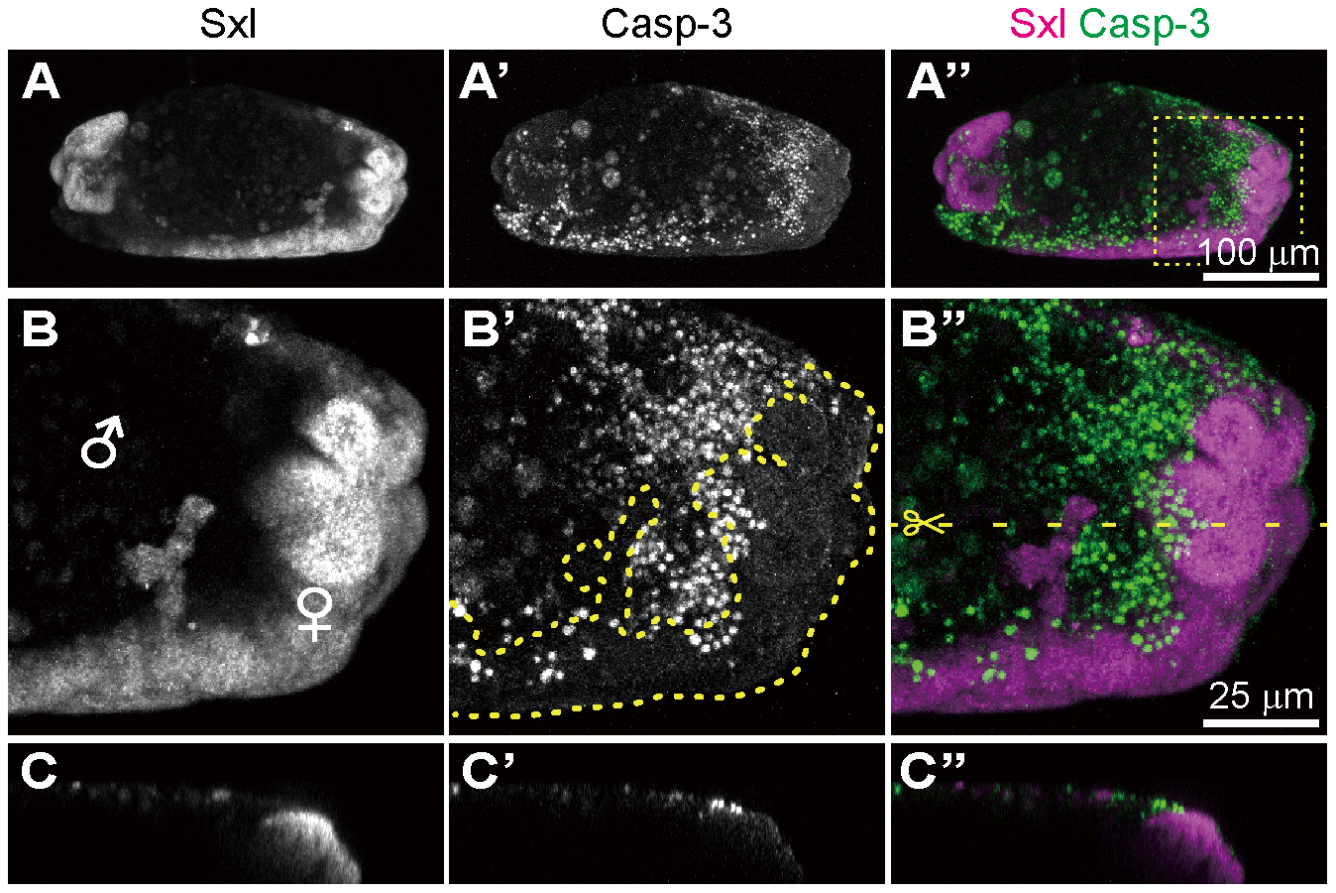
*Spiroplasma*-infected gynandromorphic embryo of *D. melanogaster* wherein apoptotic cell death is associated with male cells. This is most likely a stage 13 embryo, although its precise developmental stage cannot be determined because of severe morphological defects. (**A**–**C**) Images of immunostaining with anti-Sxl antibody by which female cells are specifically stained. (**A′**–**C′**) Images of immunostaining with anti-cleaved-Casp-3 antibody by which apoptotic cells are selectively visualized. (**A″**–**C″**) Merged images. The boxed region in (**A″**) is magnified in (**B**)–(**B″**). Z-sections of dotted yellow line in (**B″**) is shown in (**C**)–(**C″**). In (**B′**), the area of Sxl-expressing female cells is highlighted by dotted yellow line.

In the survey of *Spiroplasma*-infected embryos, we occasionally identified gynandromorphic embryos with mosaic expression of Sxl (Fig. 6). Sxl-mosaic embryos were observed at stage 12 and later (3/162; 1.9%), but not found at earlier stages (stage 9 to 11; 0/142). In *D. melanogaster*, spontaneous gynandromorphism has been reported to occur at frequencies between 0.02 to 0.1% in XX zygotes [55]. While symbiont-induced gynandromorphism has been reported from *Wolbachia*-infected moth, butterfly, planthopper, wasp and wood louse [56]–[60], it requires further verification whether or not the infrequent occurrence of gynandromorphism in *D. melanogaster* is induced by *Spiroplasma* infection. Interestingly, apoptotic cells labeled with anti-cleaved-Caspase-3 antibody were restricted to Sxl-negative, presumable male areas in the gynandromorphic embryos (Fig. 6B and C). These results provide strong evidence that *Spiroplasma* infection selectively acts on male cells but not on female cells, thereby causing male-killing phenotype.

### Conclusion and perspective

In conclusion, our study unveiled previously unknown molecular and cellular aspects underlying the *Spiroplasma*-induced male-killing in *D. melanogaster*. We demonstrated that in *Spiroplasma*-infected *Drosophila* embryos (i) host's apoptotic pathway is up-regulated in a male-specific manner, (ii) the male-specific apoptosis mainly targets embryonic epithelial cells, (iii) as previously reported, remarkable neural malformation is observed in male embryos, (iv) however, neither differentiated neural cells nor precursor neural cells exhibit apoptosis in male embryos, (v) the male-specific neural malformation occurs even when host's apoptotic pathway is disrupted, and (vi) therefore, the apoptosis-dependent epidermal cell death and the apoptosis-independent neural malformation may represent different mechanisms underlying the *Spiroplasma*-induced lethality in male embryos. We also found that (vii) *Spiroplasma* titers remain almost constant throughout the embryonic development and across male and female embryos, (viii) although at a low frequency (∼2%), gynandromorphic embryos are found in the *Spiroplasma*-infected embryos, (ix) in these embryos, apoptotic cell death is preferentially observed in male cells, and (x) therefore, neither quantity nor proliferation of *Spiroplasma* but some *Spiroplasma*-derived factor(s) selectively acting on host's male cells may be responsible for the expression of male-killing phenotype. These findings highlight complex molecular and cellular interactions in the *Spiroplasma*-*Drosophila* symbiosis, and provide invaluable clues to our deeper understanding of the symbiont-induced manipulation of host's development and reproduction.

## Materials and Methods

### Fly stocks and genetics

The following laboratory strains of *D. melanogaster* were raised at 25°C on a standard cornmeal diet in plastic tubes unless otherwise indicated. Oregon-R (wild-type strain) was provided by Takehide Murata (the Institute of Physical and Chemical Research, RIKEN). *Sxl-Pe-EGFP* G78b [61] and *Df(3L)H99, kni^ri-1^*, *p^p^*/*TM3*, *Sb^1^* [40] were obtained from the Bloomington Stock Center, USA, and the *Drosophila* Genetic Resource Center (DGRC) at Kyoto Institute of Technology, Japan, respectively. After tetracycline treatment for curing bacterial infections as described [62], the fly strains were infected with the *Spiroplasma* strain MSRO by hemolymph injection as described [16]. The MSRO-containing hemolymph was collected from naturally infected *D. melanogaster* strain Ug-SR derived from Uganda [63], which was gifted by John Jaenike (University of Rochester, USA). Since the MSRO-infected fly strains produce all-female offspring, these strains were maintained by supplying males from corresponding uninfected fly stocks. *H99* mutant strain was re-balanced with GFP-tagged balancer (*TM3, ActGFP, Ser^1^*) and homozygous mutant individuals were identified by immunostaining with anti-GFP antibody.

### Immunohistochemistry

*Spiroplasma*-infected female flies within three days after eclosion were allowed to mate with male flies for three days in plastic tubes. These insects were kept with grape juice agar plates for embryo collection. Embryos at different developmental stages were dechorionated, fixed in 4% formaldehyde and heptane for 20 min, and devitellinized by vigorously shaking in heptane and methanol. In this study, female-specific expression of *Sxl*, the master regulator of the sex determination system in *Drosophila* [64], [65], was used for sexing of embryos (Fig. S1A and B). The following primary antibodies were used for immunohistochemical staining: mouse anti-Sex-lethal (M18; 1∶20 dilution; Developmental Studies Hybridoma Bank [DSHB]), rabbit anti-cleaved-Caspase-3 (1∶100; Cell signaling Technology, Inc.), rat anti-*D*α-Catenin (DCAT-1; 1∶10; DSHB), rabbit anti-PKCζ (C-20; 1∶100; Santa Cruz Biotechnology, Inc.), mouse anti-Elav (9F8A9; 1∶20; DSHB), rat anti-Elav (7E8A10; 1∶20; DSHB), chicken anti-GFP (GFP-1020; 1∶400; Aves Labs, Inc.), mouse anti-Wingless (4D4; 1∶20; DSHB), mouse anti-Engrailed/Invected (4D9; 1∶20; DSHB), mouse anti-Antp (8C11; 1∶20; DSHB), mouse anti-Ubx (FP3.38; 1∶20; DSHB), mouse anti-Abd-B (1A2E9; 1∶20; DSHB), and guinea pig anti-Krüppel (#573; 1∶300; Asian Distribution Center for Segmentation Antibodies) [66]. Fluorochrome-labeled secondary antibodies were purchased from Jackson ImmunoResearch Laboratories, Inc. and Molecular Probes. Nuclear DNA was stained with SYTOX Orange Nucleic Acid Stain (S-11368; 1∶20,000; Molecular Probes). TUNEL staining was performed to detect DNA fragmentation associated with programmed cell death or apoptosis [67], [68] by In Situ Cell Death Detection Kit, TMR red (Roche Applied Science) as described [69]. Images were taken on a confocal microscope (Zeiss LSM 5 Pascal and LSM 510 META). Serial Z-sections of confocal images were compiled to create projection images (maximum intensity projection) unless otherwise described, using a custom macro in ImageJ software (National Institutes of Health, USA).

### Quantitative PCR

After dechorionization, developmental stages and sexes of *Sxl-Pe-EGFP* embryos were determined visually under a stereoscopic fluorescent microscope (Leica M165 FC). Each of 12 embryos of both sexes, which were collected at stage 10, 11, 12 or 13, was individually subjected to DNA extraction using QIAamp DNA mini kit (Qiagen). The DNA samples were subjected to real-time quantitative PCR using SYBR Green (Takara) and Mx3000P qPCR system (Stratagene) essentially as described [16]. *Spiroplasma* titers in terms of *dnaA* gene copies were quantified using the primers 5**′**-TGA AAA AAA CAA ACA AAT TGT TAT TAC TTC-3**′** and 5**′**-TTA AGA GCA GTT TCA AAA TCG GG-3**′** . The copy numbers of the host *EF1α* gene were also quantified using the primers 5**′**-TTA ACA TTG TGG TCA TTG GCC A-3**′** and 5**′**-CTT CTC AAT CGT ACG CTT GTC G-3**′** . The reaction mixture consisted of 1× AmpliTaq Gold buffer, 1.5 mM MgCl_2_, 0.2 mM each of dATP, dGTP, dCTP and dUTP, 0.3 µM each of the forward and reverse primers, 1/100,000 SYBR green, and 0.02 U/µl AmpliTaq Gold DNA polymerase (Applied Biosystems). PCR was performed under a temperature profile of 95°C for 10 min followed by 38 cycles of 95°C for 1 min, 60°C for 1 min and 72°C for 1 min. The data were statistically analyzed using the software R version 2.15.0 (R Foundation for Statistical Computing). Multiple comparison was performed using non-parametric Kruskal-Wallis test followed by Scheffe test.

### Imaging analysis

Quantitative analyses of TUNEL signals were performed by custom R scripts with EBImage package for image processing [70]. Briefly, maximum projections of confocal slices stained with TUNEL and Sxl antibody were obtained. Images showing lateral view of the embryos were selected for further processing. Embryonic regions were determined by binarization of projected images of Sxl. Processed images were visually checked and signals derived from other objects (e.g. flanking embryos, backgrounds etc.) were manually blacked out to obtain the area of each embryo precisely (mask image). TUNEL signals were also binarized and signals inside the mask image were calculated by image integration. For normalization, TUNEL signals in each embryo were divided by the embryonic area calculated by mask image. Statistical test was performed using Wilcoxon rank sum test.

## Supporting Information

Figure S1 **Sxl-based molecular sexing and TUNEL-based detection of apoptosis in control embryos of** ***D. melanogaster*** **.** (**A** and **B**) Female-specific EGFP signals in stage 12 embryos of the transgenic strain *Sxl-Pe-EGFP*, in which EGFP is expressed under the control of *Sxl* early promoter (Pe). (**A′** and **B′**) Female-specific immunostaining of stage 12 embryos with anti-Sxl antibody. (**A″** and **B″**) Merged images. (**C** and **D**) TUNEL staining of stage 11 control embryos. (**C′** and **D′**) Double-staining of stage 11 control embryos with TUNEL and anti-DCAT-1 antibody. (**E**) Comparison of TUNEL-positive areas between control female embryos (red) and male embryos (blue) at stage 11. Medians and interquartile ranges are shown with sample sizes. No significant difference is detected between female embryos and male embryos (Wilcoxon rank sum test; *P* = 1). (**F**) Infection dynamics of the male-killing *Spiroplasma* in developing female embryos (red) and male embryos (blue) from stage 10 to stage 13 in terms of symbiont *dnaA* gene copies per host *EF1α* gene copy. (**G**) Dynamics of host *EF1α* gene copies in developing female embryos (red) and male embryos (blue) from stage 10 to stage 13. Medians and interquartile ranges of 12 measurements are shown. Different characters show significant statistical differences (Kruskal-Wallis test followed by Scheffe test; *P*<0.05).
(TIF)

Figure S2 **Expression patterns of Hox proteins and segment polarity proteins in** ***Spiroplasma*** **-infected embryos of** ***D. melanogaster*** **.** *Spiroplasma*-infected female embryos (**A**, **C**, **E**, **G** and **I**) and male embryos (**B**, **D**, **F**, **H** and **J**) are stained with antibodies against Hox proteins Antennapedia (Antp; **A** and **B**), Ultrabithorax (Ubx; **C** and **D**) and Abdominal B (Abd-B; **E** and **F**), and segment polarity proteins Wingless (Wg; **G** and **H**) and Engrailed (En; **I** and **J**). These embryos are counter-stained for nuclear DNA (magenta in merged images).
(TIF)
