# Supplementary figures and images for: Male-Killing Spiroplasma Induces Sex-Specific Cell Death via Host Apoptotic Pathway
Source: PLoS Pathog. 2014 Feb 13;10(2):e1003956. doi: 10.1371/journal.ppat.1003956 (PMC3923752; doi:10.1371/journal.ppat.1003956)

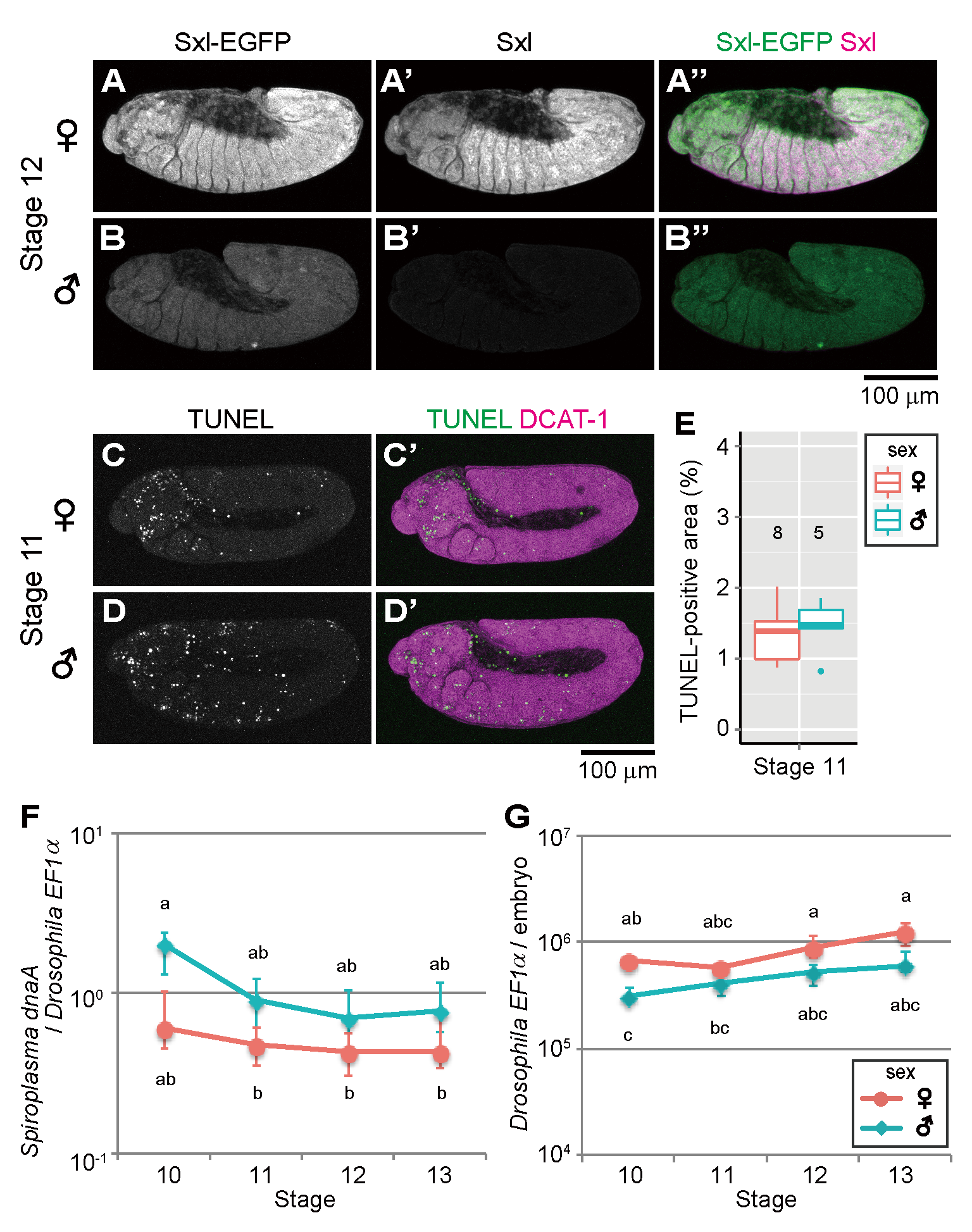

Supplement: Figure S1 — Sxl-based molecular sexing and TUNEL-based detection of apoptosis in control embryos of D. melanogaster . (A and B) Female-specific EGFP signals in stage 12 embryos of the transgenic strain Sxl-Pe-EGFP, in which EGFP is expressed under the control of Sxl early promoter (Pe). (A′ and B′) Female-specific immunostaining of stage 12 embryos with anti-Sxl antibody. (A″ and B″) Merged images. (C and D) TUNEL staining of stage 11 control embryos. (C′ and D′) Double-staining of stage 11 control embryos with TUNEL and anti-DCAT-1 antibody. (E) Comparison of TUNEL-positive areas between control female embryos (red) and male embryos (blue) at stage 11. Medians and interquartile ranges are shown with sample sizes. No significant difference is detected between female embryos and male embryos (Wilcoxon rank sum test; P = 1). (F) Infection dynamics of the male-killing Spiroplasma in developing female embryos (red) and male embryos (blue) from stage 10 to stage 13 in terms of symbiont dnaA gene copies per host EF1α gene copy. (G) Dynamics of host EF1α gene copies in developing female embryos (red) and male embryos (blue) from stage 10 to stage 13. Medians and interquartile ranges of 12 measurements are shown. Different characters show significant statistical differences (Kruskal-Wallis test followed by Scheffe test; P<0.05). (TIF) [file ppat.1003956.s001.tif]

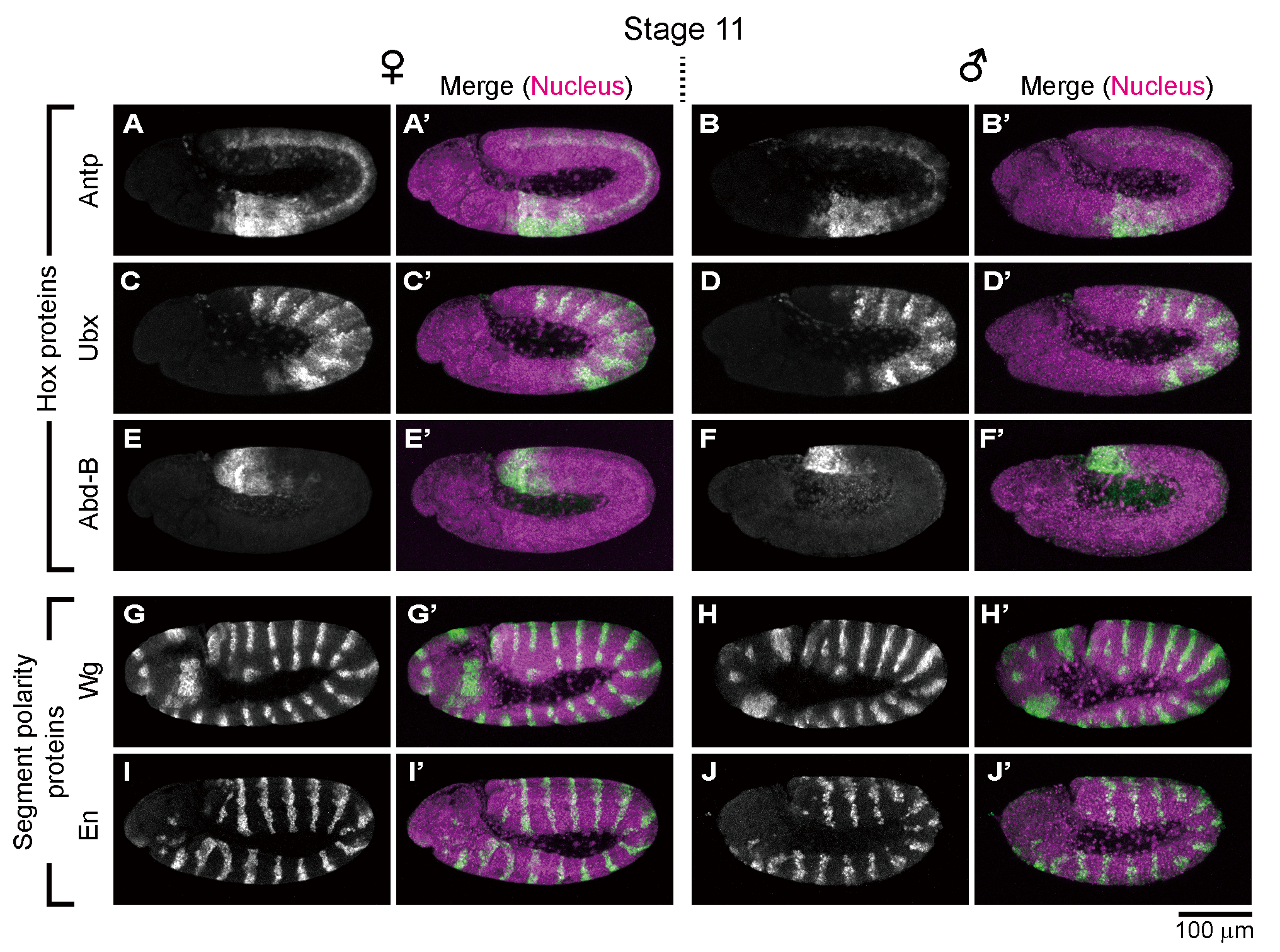

Supplement: Figure S2 — Expression patterns of Hox proteins and segment polarity proteins in Spiroplasma -infected embryos of D. melanogaster . Spiroplasma-infected female embryos (A, C, E, G and I) and male embryos (B, D, F, H and J) are stained with antibodies against Hox proteins Antennapedia (Antp; A and B), Ultrabithorax (Ubx; C and D) and Abdominal B (Abd-B; E and F), and segment polarity proteins Wingless (Wg; G and H) and Engrailed (En; I and J). These embryos are counter-stained for nuclear DNA (magenta in merged images). (TIF) [file ppat.1003956.s002.tif]
